# Brassinosteroids suppress ethylene-induced fruitlet abscission through LcBZR1/2-mediated transcriptional repression of *LcACS1*/*4* and *LcACO2*/*3* in litchi

**DOI:** 10.1038/s41438-021-00540-z

**Published:** 2021-05-01

**Authors:** Xingshuai Ma, Ye Yuan, Caiqin Li, Qian Wu, Zidi He, Jianguo Li, Minglei Zhao

**Affiliations:** 1 State Key Laboratory for Conservation and Utilization of Subtropical Agro-Bioresources, China Litchi Research Center, South China Agricultural University, 510642 Guangzhou, China; 2 Guangdong Litchi Engineering Research Center, College of Horticulture, South China Agricultural University, 510642 Guangzhou, China; 3 Ministry of Agriculture and Rural Affairs Key Laboratory of South China Horticultural Crop Biology and Germplasm Enhancement, College of Horticulture, South China Agricultural University, 510642 Guangzhou, China

**Keywords:** Plant molecular biology, Brassinosteroid

## Abstract

Abscission in plants is tightly controlled by multiple phytohormones and the expression of various genes. However, whether the plant hormone brassinosteroids (BRs) are involved in this process is largely unknown. Here, we found that exogenous application of BRs reduced the ethylene-induced fruitlet abscission of litchi due to lower ethylene (ET) production and suppressed the expression of the ethylene biosynthetic genes *LcACS1*/*4* and *LcACO2*/*3* in the fruitlet abscission zone (FAZ). Two genes that encode the BR core signaling components brassinazole resistant (BZR) proteins, namely, *LcBZR1* and *LcBZR2*, were characterized. LcBZR1/2 were localized to the nucleus and acted as transcription repressors. Interestingly, the *LcBZR1*/*2* transcript levels were not changed during ET-induced fruitlet abscission, while their expression levels were significantly increased after BR application. Moreover, gel shift and transient expression assays indicated that LcBZR1/2 could suppress the transcription of *LcACS1*/*4* and *LcACO2*/*3* by specifically binding to their promoters. Importantly, ectopic expression of *LcBZR1*/*2* in *Arabidopsis* significantly delayed floral organ abscission and suppressed ethylene biosynthesis. Collectively, our results suggest that BRs suppress ET-induced fruitlet abscission through LcBZR1/2-controlled expression of genes related to ethylene biosynthesis in litchi. In addition, similar results were observed in the *Arabidopsis* gain-of-function mutant *bzr1-1D*, which showed delayed floral organ abscission in parallel with lower expression of *ACS*/*ACO* genes and reduced ethylene production, suggesting that the mechanism of BZR-controlled organ abscission via regulation of ethylene biosynthesis might be conserved in *Arabidopsis*.

## Introduction

Abscission in plants, which occurs in a specific region called the abscission zone (AZ), is a physiological phenomenon and complex process. Abscission, in an evolutionary context, is a highly significant process that causes seed and fruit dispersal and eliminates infected or damaged organs. From an agricultural perspective, however, unexpected abscission is a key limiting factor for crop productivity. Therefore, exploring the mechanisms underlying abscission will help in management practices, such as young fruit thinning and crop production regulation^1,2^.

Organ abscission is initiated by many internal and external cues, among which plant hormones have been revealed to play major roles in regulating the initiation of abscission^2,3^. In general, ethylene (ET) and auxin are considered major regulators, and they function in an antagonistic manner to regulate abscission^4,5^. The effect of ABA and cytokinins on plant organ abscission seems to be mediated by auxin or ET rather than a direct effect^4,6–9^. Regarding brassinosteroids (BRs), to the best of our knowledge, only one report has shown that BRs delay the abscission of *Citrus* leaf and fruitlet explants^10^. However, whether BRs play a role in abscission in plants is largely unknown.

BRs are plant steroid hormones that play critical roles in growth and in responses to abiotic and biotic stresses^11^. In *Arabidopsis*, extensive studies of BR biosynthesis or signaling mutants have identified key BR signaling components, ranging from receptor kinases to downstream transcription factors (TFs)^12^. Molecular genetic studies have revealed a phosphorylation-mediated signaling pathway that controls the protein stability, subcellular localization, and transcriptional activity of two homologous TFs, namely, BRASSINAZOLE RESISTANT1 (BZR1) and BZR2, also called BRI1-EMS-SUPPRESSOR1 (BES1)^12–14^. When BRs are present at low levels or are absent, BZR1 and BES1 are phosphorylated by active BIN2^13–15^, which abolishes their DNA-binding activity and leads to their cytoplasmic retention by 14-3-3 proteins^16–18^. When BR levels are high, BZR1 and BES1, which are dephosphorylated by protein phosphatase 2A (PP2A), can transport them into the nucleus and then bind to their target gene promoters, resulting in gene activation or repression^19–22^.

As central regulators of BR signaling, BZR1/BES1 can bind to their target genes via the BR response motif (BRRE; CGTGC/TG) or the E-box element (CANNTG) to activate the BR response^20–23^. In *Arabidopsis*, a genome-wide protein-DNA interaction assay has identified more than 1000 target genes of BZR1 that are involved in specific cellular, metabolic, and developmental processes^21^. Interestingly, recent reports have revealed that BRs are involved in fruit ripening. For example, exogenous application of brassinolide (the most active BR) on a tomato plant causes rapid ripening, possibly due to the upregulation of ET biosynthetic genes such as *LeACS2*/*4* and *LeACO1*/*4*, whereas plants treated with brassinazole (a BR biosynthesis inhibitor) show delayed ripening and suppressed expression of these ET biosynthetic genes^24^. Similarly, persimmon fruit treated with 2,4-epibrassinolide (EBR) exhibited rapid fruit softening and upregulation of ET biosynthetic genes, including *DkACO2* and *DkACS1*/*2*. In contrast, brassinazole treatment delayed persimmon fruit ripening^25^. Together, these results suggest that BRs have a role in ET biosynthesis during fruit ripening.

Litchi (*Litchi chinensis* Sonn.) is a vital tropical fruit crop, but it exhibits severe precocious fruit abscission^26,27^. Our previous reports demonstrated that ET is the major inducer of litchi fruitlet abscission, and *LcACS1*/*4* and *LcACO2*/*3* play dominant roles in ET production during abscission^28,29^. In addition, we established a system using two abscission-accelerating treatments, including the application of ethephon to induce litchi fruitlet abscission^28–30^. Based on these findings, we screened for effective chemicals to control litchi fruitlet abscission by regulating the biosynthesis of ET. We found that exogenous application of BRs repressed ET production and therefore reduced ethephon (ET)-induced fruitlet abscission in litchi. Importantly, we propose that two BZR homologs, i.e., LcBZR1/2, negatively regulate fruitlet abscission by directly repressing the expression of *LcACS1*/*4* and *LcACO2*/*3*.

## Results

### Exogenous application of BRs represses ET-induced ethylene production, fruitlet abscission, and the expression of *LcACS1*/*4* and *LcACO2*/*3* in litchi

**Fig. 1:**
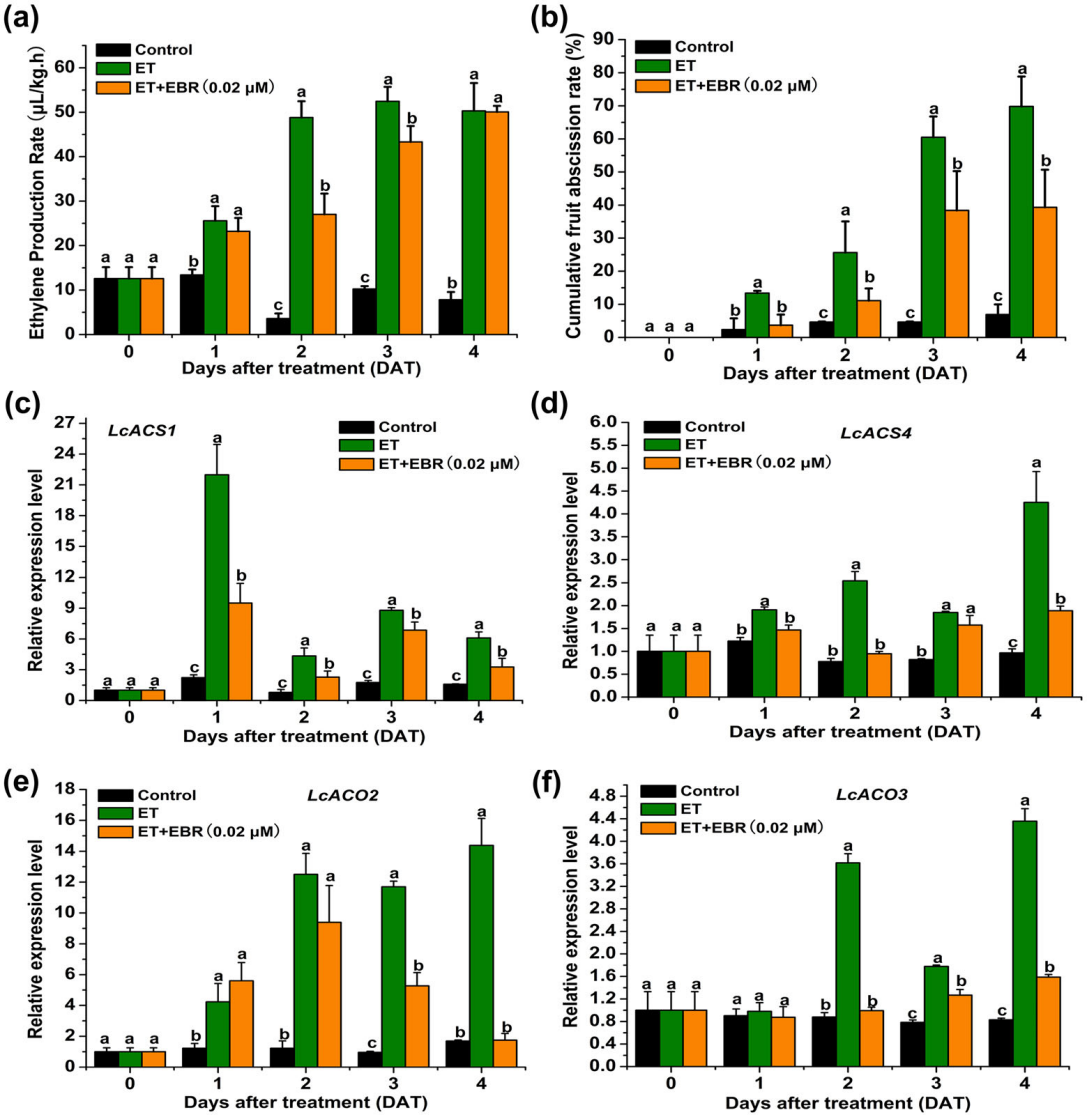
EBR suppresses ethylene-induced fruit abscission and the expression of *LcACS1/4* and *LcACO2/3* in litchi. **a** Ethylene production in fruitlets after ethylene (ET) or ethylene plus 2,4-epibrassinolide (ET + EBR) treatment. **b** Cumulative abscission rate of fruitlets after ET or ET + EBR treatment. **c**–**f** qRT-PCR analysis of *LcACS1*/*4* and *LcACO2*/*3* expression at the fruitlet abscission zone after ET or ET + EBR treatment. Letters indicate that the differences between the control and treatments were significant (*p* < 0.05).

To determine the effect of BRs on ET-induced fruitlet abscission in litchi, we treated the fruitlets with ethephon alone (ET) or in combination with 24-epibrassinolide at a concentration of 0.02 µM (ET + EBR). As shown in Fig. 1a, ET production in fruitlets was induced on the second day after ethephon treatment and peaked 3 days after ethephon treatment (DAT). In contrast, when ethephon and EBR treatments were combined, ET production was greatly repressed at two and three DAT (Fig. 1a). As a result, the cumulative fruitlet abscission rate (CFAR) was significantly reduced by ET + EBR treatment compared with ET treatment alone (Fig. 1b). At four DAT, 69.8% of the fruitlets dropped under ET treatment, whereas 39.3% of the fruitlets dropped under ET + EBR treatment (Fig. 1b). All fruitlets dropped at five DAT under ET treatment, but 46.25% of the fruitlets remained under ET + EBR treatment. Our previous studies demonstrated that *LcACS1*/*4* and *LcACO2*/*3* play dominant roles in ET-induced ET production^28,29^; therefore, we tested their expression levels under ET + EBR treatment. We found that the ET-induced expression levels of *LcACS1*/*4* and *LcACO2*/*3* were dramatically repressed by ET + EBR treatment in the fruitlet abscission zone (FAZ) (Fig. 1c–f), which is consistent with ET production under the ET and ET + EBR treatments.

### Identification and characterization of two BZR homologs, LcBZR1 and LcBZR2, in litchi

**Fig. 2:**
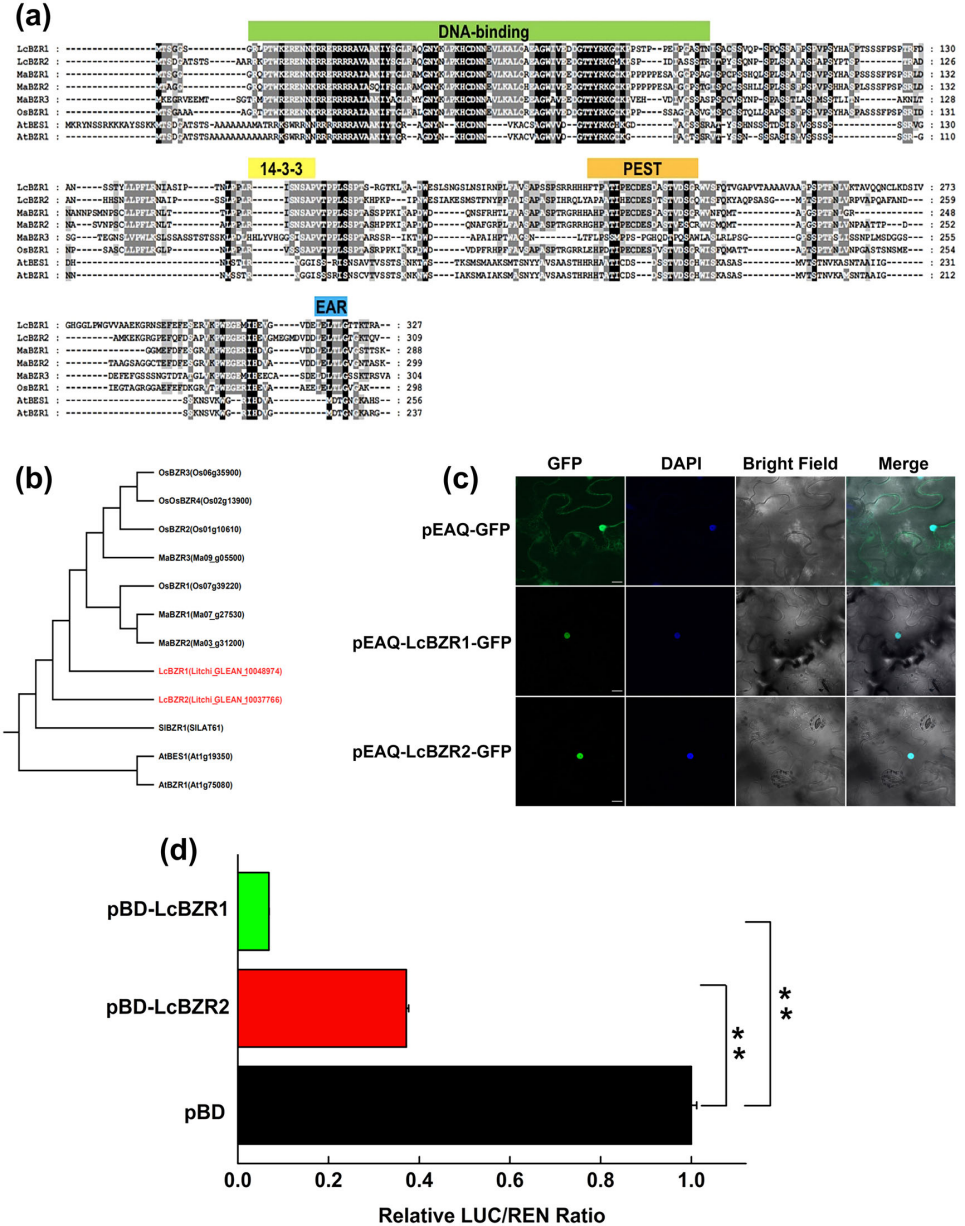
Characterization of LcBZR1 and LcBZR2. **a** Amino acid sequence alignment of LcBZR1/2 with homologs from banana (Ma, *Musa acuminate* L.), rice (Os, *Oryza sativa* L.) and Arabidopsis (At, *Arabidopsis thaliana*). **b** Phylogenetic analysis of LcBZR1/2 with homologs from banana (Ma, *Musa acuminate* L.), rice (Os, *Oryza sativa* L.), tomato (Sl, *Solanum lycopersicum*) and Arabidopsis (At, *Arabidopsis thaliana*). **c** Subcellular localization of LcBZR1/2 in tobacco leaves. LcBZR1/2 fused to GFP and the positive control pEAQ-GFP were transformed into tobacco leaves. After 48 h of incubation, GFP signals were observed under a confocal laser scanning microscope. The nucleus is indicated by 4,6-diamidino-2-phenylindole (DAPI) staining. Merged images indicate the colocalization of DAPI and GFP signals. Scale bars indicate 25 µm. **d** Transcriptional repression ability of LcBZR1/2 in tobacco leaves. The transcriptional activity of LcBZR1/2 was tested by the dal-luciferase reporter assay by detecting the ratio of LUC to REN. The SE was calculated from six replicates. **Significant differences in values according to Student’s *t* test (*P* < 0.01).

To identify the BZR homologs in litchi, we manually searched for BZR genes using TBLASTN against the litchi genome (http://111.230.180.7:81/index.php) with TBtools^31^. Two BZR-like genes were identified and were named *LcBZR1* and *LcBZR2*. Amino acid sequence alignment revealed that LcBZR1/2 contained the typical features of BZR TFs, including a DNA-binding domain, a 14-3-3 binding region (RISNSAP), and a putative PEST sequence (Fig. 2a). In addition, LcBZR1/2 contained the EAR (ET-responsive element binding factor-associated amphiphilic repression) region (LxLxLx) near the C-terminus, which implies that they might act as transcriptional repressors (Fig. 2a). Furthermore, a phylogenetic tree was constructed using LcBZR1/2 and other BZR TF sequences from *Arabidopsis*, tomato plants, rice, and banana plants, which indicated that LcBZR1/2 were not closely grouped with these BZR TFs (Fig. 2b), suggesting that LcBZR1/2 found in the litchi genome might be novel members of the BZR transcription factor family.

To investigate the subcellular localization of LcBZR1/2, the open reading frame sequences of *LcBZR1*/*2* were subcloned into a green fluorescent protein (GFP)-tagged vector (pEAQ). Transient expression of these constructs in *N. benthamiana* leaf epidermal cells showed that the fluorescence of pEAQ–LcBZR1–GFP or pEAQ–LcBZR2–GFP was predominantly localized in the nucleus. In contrast, GFP signals of the pEAQ–GFP control were uniformly observed throughout the cell (Fig. 2c).

It has been reported that TFs containing an EAR domain function as transcription repressors^32,33^. To validate the transcriptional activity of LcBZR1/2, we performed a transient expression assay using a dual-luciferase reporter system and coexpressed the reporter with an effector consisting of LcBZR1 or LcBZR2 in *N. benthamiana* leaf epidermal cells. We observed a significantly lower LUC/REN ratio when the reporter was coexpressed with pBD-LcBZR1 or pBD-LcBZR2 compared with the pBD control (Fig. 2d), indicating that both LcBZR1 and LcBZR2 are transcriptional repressors.

### LcBZR1/2 directly binds to and represses the promoter activity of *LcACS1*/*4* and *LcACO2*/*3*

**Fig. 3:**
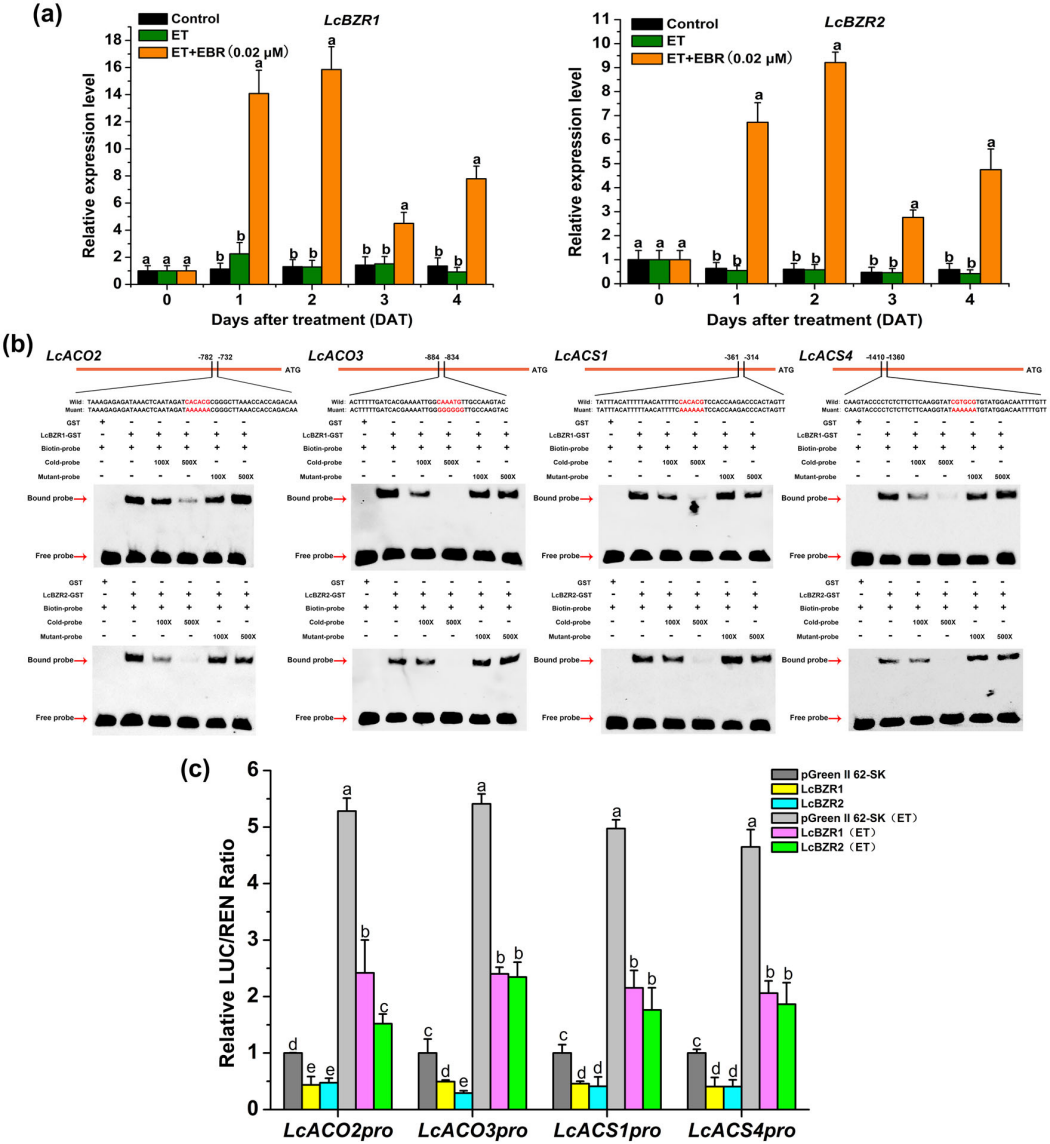
Binding of LcBZR1/2 to the *LcACO2/3* and *LcACS1/4* promoters to repress their expression. **a** *LcBZR1*/*2* were induced in the fruitlet abscission zone (FAZ) under ET + EBR treatment. **b** Electrophoretic mobility shift assays (EMSAs) showing the binding ability of LcBZR1/2 with the promoters of *LcACO2/3* and *LcACS1/4* in vitro. Sequences of both the mutant and wild-type probes are presented on the top. Shifted bands, indicating the formation of DNA-protein complexes, are marked by arrows. ‘+’ and ‘-’ represent presence and absence, respectively. 100× and 500× indicate increasing amounts of mutant or unlabeled probes used for testing the specificity of binding and competition. Probes without biotin labels were loaded as unlabeled competitors. GST protein alone was used as the negative control. **c** LcBZR1/2 suppressed the expression of *LcACO2/3* and *LcACS1/4* in vivo, as shown by transient dual-luciferase reporter assays. Both effector and reporter vectors were cotransformed into tobacco leaves. After incubation with or without ethylene (ET, 50 µl L^−1^) for 48 h the ratio of LUC to REN was detected. Error bars indicate SEs from six replicates. Different letters indicate a significant difference (*p* < 0.05).

To study the possible role of LcBZR1/2 in BR-suppressed, ET-induced fruitlet abscission in litchi, we examined their expression patterns at the FAZ using qRT-PCR. We found that *LcBZR1*/*2* transcripts did not change in either the control or ET-treated FAZ tissues, whereas their expression levels in the FAZ were significantly increased after the combination of ET and EBR treatments (Fig. 3a), indicating that *LcBZR1*/*2* are involved in BR-suppressed fruitlet abscission in litchi. As central regulators of BR signaling, previous findings have revealed that BZR proteins can target ET biosynthetic genes to regulate ET production^34,35^. We thus hypothesized that LcBZR1/2 might be involved in BR-suppressing, ET-induced fruitlet abscission by regulating ET biosynthesis. To test this hypothesis, we screened for the *cis* elements that are responsible for the binding of BZR TFs in the promoters of *LcACS1*/*4* and *LcACO2*/*3*. Interestingly, we found the BRRE element (CGTGC/TG) in the promoters of *LcACS1*/*4* and *LcACO2*, as well as an E-box element (CAGGTG) in the *LcACO3* promoter (Fig. 3b). We then used electrophoretic mobility shift assays (EMSAs) to investigate whether LcBZR1/2 could directly bind to the promoters of *LcACS1*/*4* and *LcACO2*/*3* in vitro. EMSAs showed that both the LcBZR1–GST and LcBZR2–GST fusion proteins were able to bind to the promoters of *LcACS1*/*4* and *LcACO2*/*3*; no such binding was detected for the GST protein alone. Furthermore, the binding to the biotin-labeled sequences was greatly reduced by unlabeled competitor sequences (cold probes) but not by mutant competitors (mutant probes) (Fig. 3b).

To examine whether LcBZR1/2 could repress these target genes, we conducted in vivo dual-luciferase reporter assays. A pGreenII 0800 vector containing an LUC reporter gene driven by the *LcACS1*/*4* or *LcACO2*/*3* promoter was cotransformed with an empty pGreenII 62-SK vector or pGreenII 62-SK-LcBZR1/2 vector into *N. benthamiana* leaf epidermal cells. Compared with the empty vector samples, the cells expressing LcBZR1 or LcBZR2 exhibited a significantly lower LUC/REN ratio (Fig. 3c). In addition, it was found that the promoter activity of *LcACS/ACO* was increased in the presence of ET. However, the cells expressing LcBZR1 or LcBZR2 still showed a significantly lower LUC/REN ratio than the empty vector control samples. Collectively, these findings support the notion that LcBZR1/2 act as transcriptional repressors of *LcACS1*/*4* and *LcACO2*/*3* by directly binding to their promoters.

### Ectopic expression of *LcBZR1*/*2* in *Arabidopsis* delays floral organ abscission

**Fig. 4:**
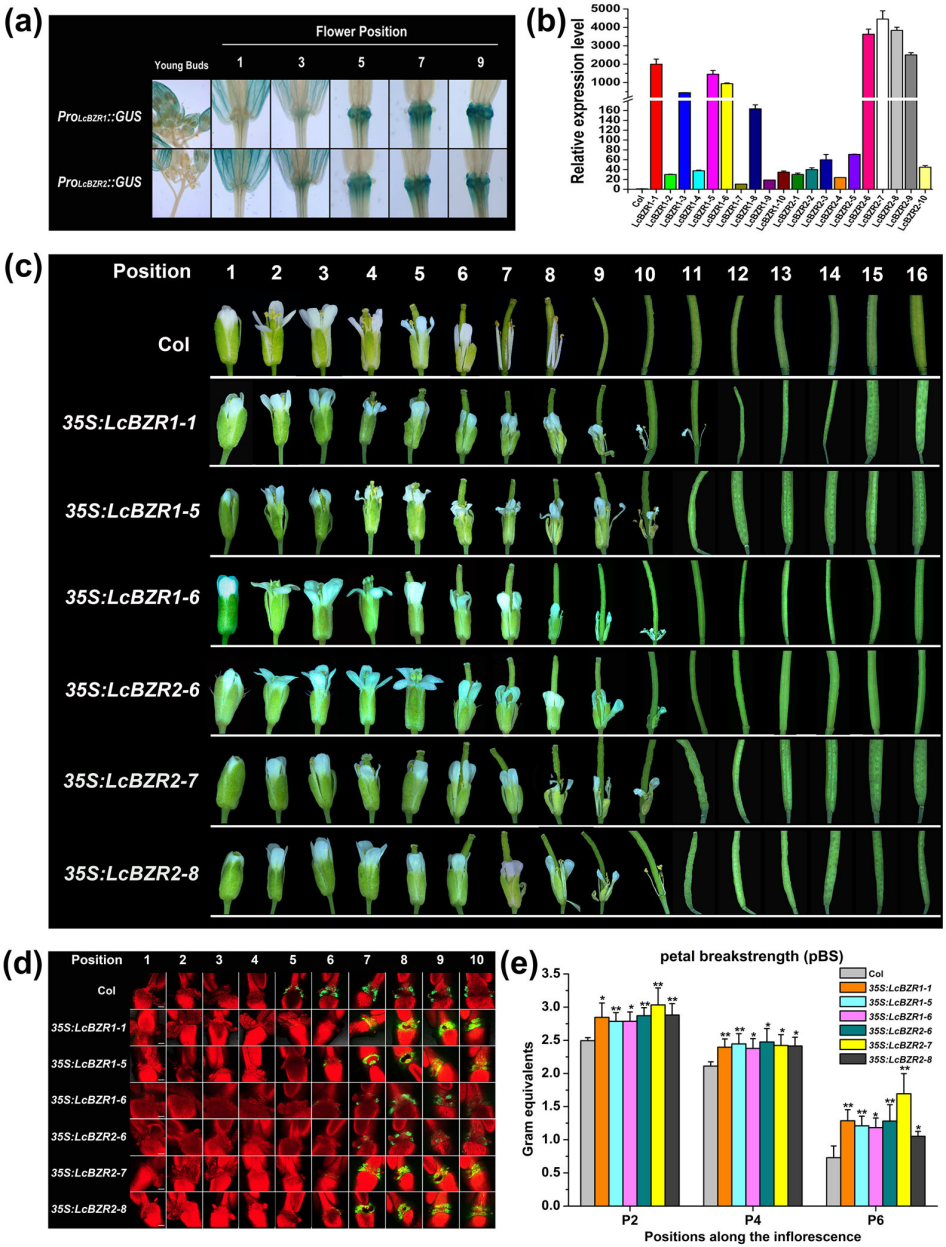
Ectopic expression of LcBZR1/2 in *Arabidopsis* delays floral organ abscission. **a** GUS expression driven by *LcBZR1*/2 promoters occurred predominantly at the floral organ abscission zone starting from position 5 in *Arabidopsis*. **b** Expression levels of *LcBZR1/2* in different transgenic lines. *LcBZR1/2* driven by the CaMV 35 S promoter was transformed into wild-type *Arabidopsis* Col. **c** Photographs of the floral organ abscission process in the Col and *LcBZR1/2* transgenic lines. Numbers on the top indicate floral positions along the inflorescence. **d** BCECF signals of the floral organ AZ of the Col and *LcBZR1/2* transgenic lines. The fluorescence images represent merged images of chlorophyll autofluorescence with BCECF fluorescence that were obtained under a confocal laser scanning microscope. The increase in pH is indicated by green fluorescence. The scale bar indicates 200 μm. The images presented for each position and plant are representative images for 3–4 replicates. **e** pBS of *LcBZR1/2* transgenic lines compared with Col (*n* = 30, bars = SD).

To further explore the functions of LcBZR1/2 in abscission, we investigated the effect of LcBZR1/2 on floral organ abscission in *Arabidopsis*. First, we observed that obvious GUS signals driven by the *LcBZR1* or *LcBZR2* promoter began to accumulate at the floral AZ of position-5 flowers in *Arabidopsis*, indicating a possible role of LcBZR1/2 in floral organ abscission in *Arabidopsis* (Fig. 4a). Next, we generated ten independent transgenic lines each for *LcBZR1* and *LcBZR2* (Fig. 4b). In wild-type Col, the floral organs, including sepals, stamens, and petals, started to drop fully at position 9. In contrast, we observed that the transgenic *Arabidopsis* lines *35* *S:LcBZR1-1*, *35* *S:LcBZR1-5*, *35* *S:LcBZR1-6*, *35* *S:LcBZR2-6*, *35* *S:LcBZR2-7*, and *35* *S:LcBZR2-8*, which showed relatively high expression levels of *LcBZR1* and *LcBZR2* (Fig. 4b), showed delayed floral organ abscission (Fig. 4c and S1). The *35* *S:LcBZR1-5*, *35* *S:LcBZR1-6*, *35* *S:LcBZR2-6*, *35* *S:LcBZR2-7*, and *35* *S:LcBZR2-8* transgenic lines dropped the full floral organs at position 11, whereas *35* *S:LcBZR1-1* plants abscised the entire floral organs at position 12 (Fig. 4c).

Previous studies revealed that floral organ abscission in *Arabidopsis* is positively correlated with the pH value in the cytoplasm of AZ cells, which can easily be detected by BCECF-AM staining^36,37^. Consistent with this, obvious BCECF signals of the floral AZ were first observed at position 7 in *35* *S:LcBZR1-1*, *35* *S:LcBZR1-5*, *35* *S:LcBZR1-6*, *35* *S:LcBZR2-6*, *35* *S:LcBZR2-7*, and *35* *S:LcBZR2-8* plants, whereas the BCECF signals were first detected at position 5 in Col plants (Fig. 4d). In addition, the delay of floral organ abscission in these transgenic lines was further quantified by petal breakstrength (pBS) measurements. As shown in Fig. 4e, before abscission, *35* *S:LcBZR1-1*, *35* *S:LcBZR1-5*, *35* *S:LcBZR1-6*, *35* *S:LcBZR2-6*, *35* *S:LcBZR2-7*, and *35* *S:LcBZR2-8* plants required more force than Col to remove petals from flowers at positions 2, 4, and 6. Collectively, these data strongly indicate that ectopic expression of *LcBZR1*/*2* in *Arabidopsis* delayed floral organ abscission.

### Ectopic expression of *LcBZR1/2* in *Arabidopsis* results in lower expression levels of *ACS*/*ACO* genes and reduced ethylene production

**Fig. 5:**
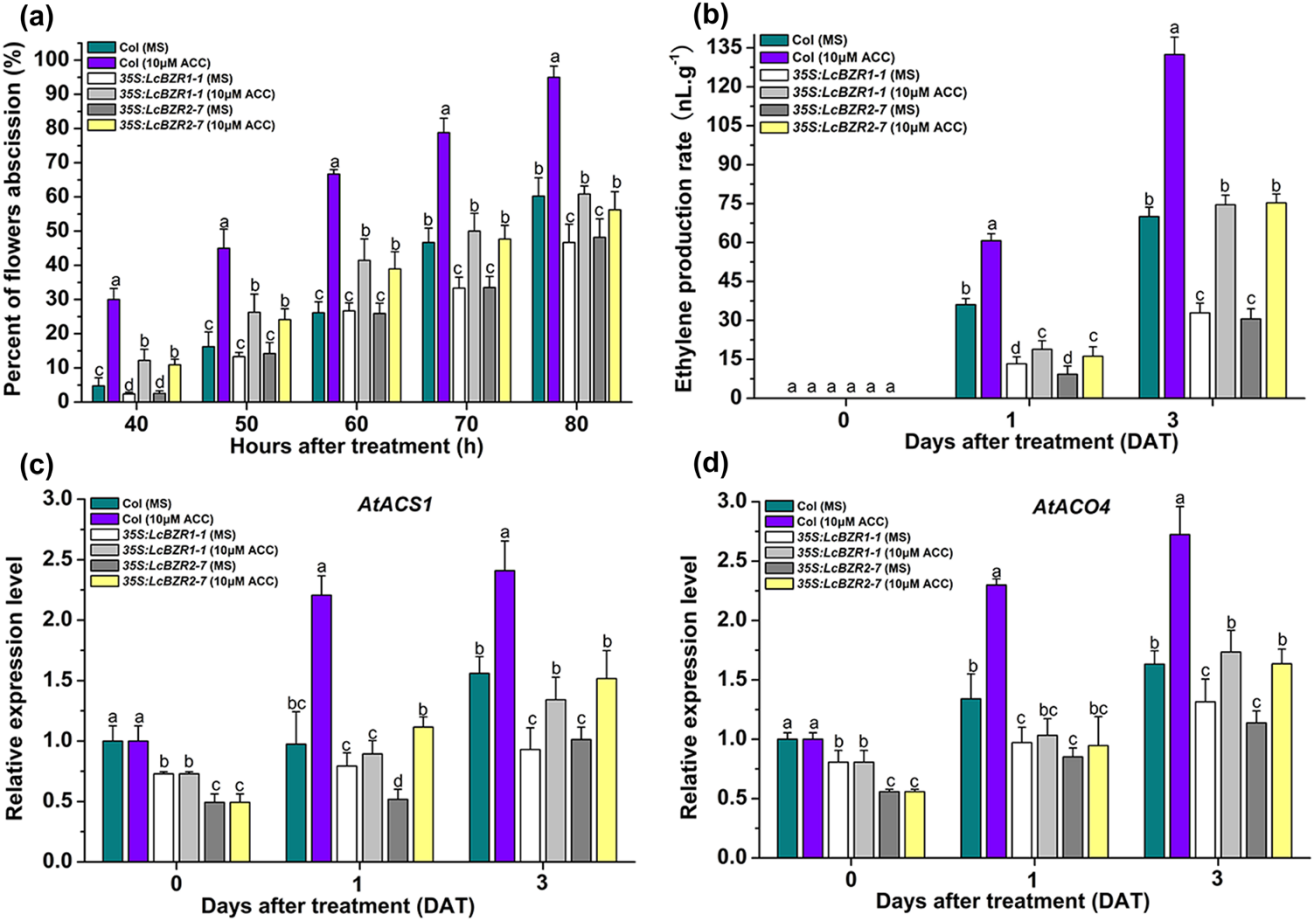
Flowers of LcBZR1/2 transgenic lines show reduced ethylene production and expression levels of *AtACS1* and *AtACO4* during floral organ abscission. **a** Flowers of Col, *35* *S:LcBZR1-1*, and *35* *S:LcBZR2-7* were cultured in MS and 10 μM ACC plates. The floral organ abscission rate was scored every 10 h. **b** Ethylene production in flowers cultured in MS or 10 μM ACC plates was detected. **c**–**d** qRT-PCR analysis of *AtACS1* and *AtACO4* expression in Col and LcBZR1/2 transgenic lines under ACC treatment. The standard error (SE) was calculated from three replicates. Different letters indicate significant pairwise differences according to Duncan’s test (*p* < 0.05).

To better understand the mechanisms of action of LcBZR1/2 in abscission in relation to ET biosynthesis, we further explored the abscission rate and the expression of ET biosynthetic genes in Col, *35* *S:LcBZR1-1* and *35* *S:LcBZR2-7* plants under treatment with 1-aminocyclopropane-1-carboxylic acid (ACC; the ET biosynthesis precursor). For this, flowers with just-visible white petals were excised from the inflorescences and then cultured on ACC plates for 4 days. As shown in Fig. 5a, *35* *S:LcBZR1-1* and *35* *S:LcBZR2-7* plants on MS plates showed a significantly greater delay in floral organ abscission than Col plants. In addition, although higher frequency abscission was observed for flowers of *35* *S:LcBZR1-1* and *35* *S:LcBZR2-7* cultured on ACC plates than for those cultured on MS plates, the frequency of abscission was significantly lower than that of Col plants cultured on the same plates. In addition, we also found that ET production by flowers from *35* *S:LcBZR1-1* and *35* *S:LcBZR2-7* plants cultured on both MS and ACC plates was partially suppressed compared with that of flower from Col plants (Fig. 5b). Furthermore, the expression levels of 15 *AtACS*/*ACO* genes (*AtACO1–4* and *AtACS1–11*) in these flowers were examined. We found that *AtACS1* and *AtACO4* were repressed in *35* *S:LcBZR1-1* and *35* *S:LcBZR2-7* plants, and their expression patterns were consistent with the floral organ abscission rates and ET production for plants cultured on MS and ACC plates (Fig. 5c, d). Taken together, these findings indicate that LcBZR1/2 suppresses the expression of *AtACS1* and *AtACO4*, thereby reducing ET production to repress floral organ abscission in *Arabidopsis*.

### The *Arabidopsis* gain-of-function mutant *bzr1-1D* showed delayed floral organ abscission

**Fig. 6:**
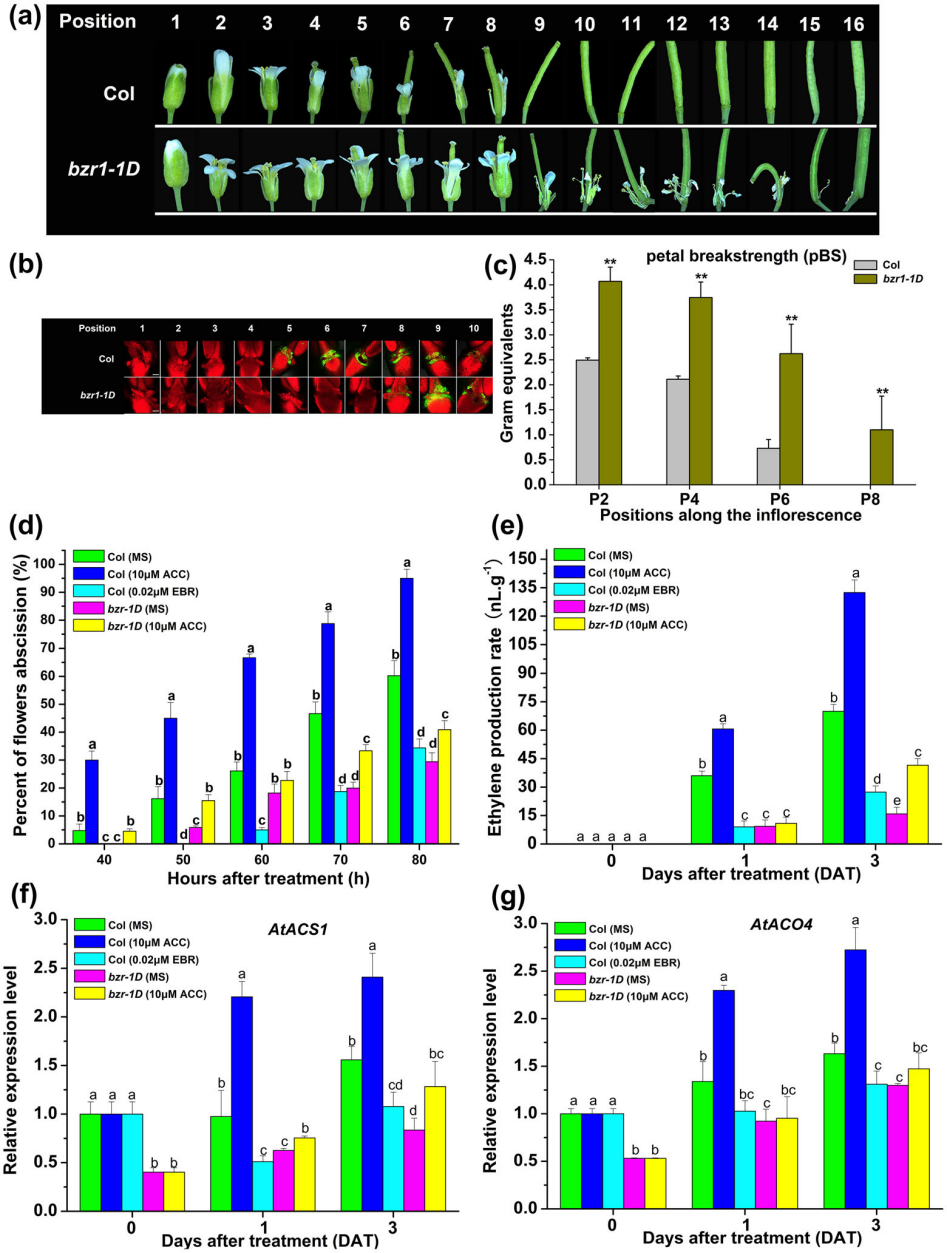
The *Arabidopsis* gain-of-function mutant *bzr1-1D* shows delayed floral organ abscission. **a** Photographs of the floral organ abscission process in Col and the *bzr1-1D* mutant. **b** BCECF signals of the floral organ AZ of Col and the *bzr1-1D* mutant. **c** pBS of the *bzr1-1D* mutant compared with that of Col (*n* = 30, bars = SD). **d** Floral organ abscission rate of Col and the *bzr1-1D* mutant under EBR or ACC treatment. **e** Ethylene production of Col and the *bzr1-1D* mutant under EBR or ACC treatment. **f**–**g** Expression levels of *AtACS1* and *AtACO4* in Col and the *bzr1-1D* mutant under EBR or ACC treatment. The SE was calculated from three replicates. Different letters indicate significant pairwise differences by Duncan’s test (*p* < 0.05).

Given that ectopic expression of *LcBZR1/2* in *Arabidopsis* delayed floral organ abscission in parallel with reduced ET production, we hypothesized that the BZR homolog in *Arabidopsis* might play a similar role in the control of floral organ abscission. To test this hypothesis, we examined the floral organ abscission process of *bzr1-1D*, a gain-of-function mutant of BZR1 involved in BR signaling^14^, in response to separate EBR and ACC treatments. First, we observed that floral organ abscission was delayed in the gain-of-function mutant *bzr1-1D*, which retained the floral organs at position 14 (Fig. 6a and S1). Consistent with this, the BCECF signals that first appeared at a later stage (position 8) in *bzr1-1D* were detected (Fig. 6b). Furthermore, at positions 2, 4, 6, and 8 before abscission, *bzr1-1D* had a higher pBS than Col (Fig. 6c). These findings demonstrate that BZR1 has a role in suppressing floral organ abscission in *Arabidopsis*. Next, detached flowers of Col and *bzr1-1D* were cultured on plates supplemented with EBR or ACC for 4 days. As shown in Fig. 6d, the floral organ abscission rate of Col was greatly reduced on EBR plates in contrast to that on MS plates. Consistent with this, *bzr1-1D* showed a lower floral organ abscission rate than Col on MS plates. When treated with ACC, *bzr1-1D* still showed a lower floral organ abscission rate than Col. In addition, we also found that ET production in flowers from *bzr1-1D* plants cultured on ACC plates was partially suppressed compared with that in flowers from Col plants (Fig. 6e). Furthermore, the expression of *AtACS1* and *AtACO4* was also repressed in *bzr1-1D* plants (Fig. 6f, g). Collectively, these results indicate that BRs suppress floral organ abscission in *Arabidopsis* through BZR1-mediated repression of ET biosynthesis.

## Discussion

As steroidal plant hormones, BRs function in a wide range of physiological and developmental events, such as seed germination, flowering, and hypocotyl elongation^12^. However, little is known about whether BRs can have any effect on plant organ abscission. In the present study, we found a biological interaction between BRs and ET production in the regulation of fruitlet abscission in litchi. This is mediated by a direct interaction between two core BR signaling TFs, i.e., LcBZR1/2, and ET biosynthetic genes.

To date, only one study has shown that BRs have a negative effect on the abscission of leaf and fruitlet explants in *Citrus*^10^. In addition, two regulatory genes involved in BR signaling, namely, BRI (BR-insensitive 1) and BAK1 (BRI1-associated receptor-like kinase 1), have been revealed to regulate floral organ abscission in *Arabidopsis*^38,39^. However, the relationship between BRs and organ abscission remains unclear. Herein, we showed that application of BRs reduced fruitlet abscission in litchi (Fig. 1b). Furthermore, the repressive role of BRs in organ abscission was verified in *Arabidopsis* (Fig. 6). These findings indicate that BRs have a negative effect on organ abscission in plants.

Previous studies have demonstrated that BRs act as positive regulators of ET biosynthesis to promote fruit ripening in climacteric fruits, including banana^34^, tomato^24^, and persimmon^25^. In the present study, we showed that BRs function as negative regulators of ET biosynthesis to suppress fruitlet abscission in litchi, a nonclimacteric fruit. Whether the effect of BRs on ET biosynthesis is dependent on fruit type requires more detailed study; after all, fruit ripening and organ abscission are different physiological processes. More importantly, although banana, tomato, and persimmon are climacteric fruits, they have evolved different types of transcriptional feedback circuits to control ET-dependent fruit ripening^24,25,34^. It has been revealed that tomato has an NAC positive feedback loop that controls ET-dependent ripening, and banana possesses both NAC and MADS positive feedback loops—a dual-loop system—to control ET-dependent ripening^40^.

Interestingly, one study in *Arabidopsis* proposed that BRs at a low concentration negatively control ET production, whereas BRs at high concentrations positively regulate ET production^35^. Ethylene production was significantly reduced in seedlings treated with a low concentration of BRs (<0.1 μM), whereas it was greatly increased when the concentration of BRs was higher than 0.5 μM^35^. Similarly, the concentration of BRs used to accelerate ET production in tomato is 5 μM^24^; in persimmon, it is 10 μM^25^; and in banana, it ranges from 1 to 4 μM^34^. In contrast, our findings showed that 0.02 μM EBR significantly repressed ET production during fruitlet abscission in litchi (Fig. 1a). Based on these findings, it seems that a low concentration of BRs (<0.1 μM) represses ET biosynthesis, while a high concentration of BRs (more than 0.5 μM) promotes ET biosynthesis. However, we cannot conclude that BRs either positively or negatively regulate ET biosynthesis in a dose-dependent manner, since the effects of different levels of BRs on ET biosynthesis are absent in these fruits. In the future, it will be of interest to determine whether exogenous application of higher concentrations of BRs could accelerate ET production, thereby promoting fruitlet abscission in litchi. This will help to determine whether the effect of BRs on ET biosynthesis is fruit type-dependent or dose-dependent.

As central regulators, the TFs BZR1/BES1 play an essential role in mediating pleiotropic BR responses by testing the specificity of BR actions by binding to various target genes^14,15^. In the present study, we provided direct evidence showing that two transcription repressors, i.e., LcBZR1/2, can bind to the BRRE motif existing in the promoters of *LcACS1/4* and *LcACO2*/*3*—which are the essential genes required for ET biosynthesis during litchi fruitlet abscission^28,29^—and can suppress their transcription (Fig. 3b, c). Our results are consistent with previous findings in *Arabidopsis*, in which BZR1/BES1 bound to the promoters of *ACS7*/*9*/*11* directly to suppress their expression^35^. Furthermore, in a recent study, MaBZR1/2 were also reported to act as transcriptional repressors of *MaACS1* and *MaACO13*/*14* during banana fruit ripening^34^. Together, these findings support the notion of the negative impact of BRs on ET production through the BZR-controlled expression of the genes involved in ET biosynthesis.

Importantly, we provide strong evidence showing that LcBZR1/2 act as repressors to control floral organ abscission in *Arabidopsis*, which is consistent with the fact that LcBZR1/2 are expressed in the floral AZ of *Arabidopsis* (Fig. 4). Interestingly, a similar role of the BZR homolog in *Arabidopsis* was also validated, as the gain-of-function mutant *bzr1-1D* delayed floral organ abscission (Fig. 6). Furthermore, two ET biosynthetic genes (*AtACS1* and *AtACO4*) and ET production were suppressed in transgenic lines overexpressing LcBZR1/2 and in the *bzr1-1D* mutant (Figs. 5 and 6), further supporting our hypothesis that BRs negatively regulate ET biosynthesis to repress abscission through BZR-mediated transcriptional repression of ET biosynthetic genes.

## Materials and methods

### Plant materials and treatments

Three 17-year-old ‘Feizixiao’ litchi trees (*Litchi chinensis* Sonn.) were selected randomly for ethephon and epibrassinolide treatment. Thirty fruitlets bearing shoots of similar diameters were chosen. For ET treatment, ten shoots were treated with 250 mg L^–1^ ethephon solution for 1 min; for ET + EBR treatment, ten shoots were dipped in 250 mg L^–1^ ethephon and 0.02 µM epibrassinolide solution for 1 min; and ten control shoots were treated with water. Determination of the CFAR and ET production in fruit were carried out as previously described^28^.

### Gene isolation, sequence analysis, and expression analysis (qRT-PCR)

The genes used in this study were retrieved from the litchi genome database (http://111.230.180.7:81/index.php), the plant genomics database (Phytozome version 12.1, https://phytozome.jgi.doe.gov/pz/portal.html), and the *Arabidopsis* genome resource (https://www.arabidopsis.org/index.jsp). Multiple sequence alignment of the BZRs was carried out by ClustalW (version 1.83) and GeneDoc software^41^. A phylogenetic tree was created in MEGA 5 with a Poisson correction model using neighbor-joining (NJ) analysis^42^.

The total RNA from the litchi fruit AZ or *Arabidopsis* flowers was extracted using a Column Plant RNAout Kit (TIANDZ, Beijing). qRT-PCR assays were carried out on a CFX96 Real-Time PCR System (Bio-Rad, Hercules, CA, USA) following the manufacturer’s instructions. *LcEF-1α*^43^ and *ubiquitin 10* (*AtUBQ*) were used as the internal reference genes for litchi and *Arabidopsis*, respectively. All reactions were carried out in triplicate.

### Subcellular localization analysis

The full-length cDNA of *LcBZR1* and *LcBZR2* was cloned into pEAQ vectors (separately) to generate pEAQ–LcBZR1/2–GFP. The fusion plasmid and pEAQ–GFP were delivered into separate tobacco (*N. benthamiana*) leaves. The transformed tobacco leaves were stained with 4,6-diamidino-2-phenylindole (DAPI). The fluorescence signal was detected under a confocal laser scanning microscope (LSM 7 DUO, ZEISS, Germany).

### Generation of transgenic plants and BCECF fluorescence assay

The overexpression vectors *35* *S:LcBZR1* and *35* *S:LcBZR2* were generated by cloning the CDSs of LcBZR1 and LcBZR2 into the vector pCAMBIA1302. Transgenic *Arabidopsis* (*Arabidopsis thaliana*) plants were obtained according to the floral dip transformation method^44^ and were then used for further phenotypic analysis and BCECF fluorescence assays^36^. Briefly, *Arabidopsis* flowers were dipped into a BCECF-AM (B1150, Thermo Scientific) working solution (10 µM) under darkness for 20 min. Then, the excess BCECE-AM was removed by phosphate-buffered saline (PBS; pH 7.4). Fluorescence images were captured using a ZEISS LSM 7 DUO confocal laser scanning microscope.

### Petal breakstrength measurement

The pBS was quantified as the force in gram equivalents required for pulling a petal from flower^45^, as determined by using a digital force gauge (Model: HF-2, Lunjie Electromechanical Instrument Co., Ltd., Shanghai). A total of 30 petals per position were measured.

### Histochemical GUS assays

The promoter regions of *LcBZR1* and *LcBZR2* were separately cloned and fused into pCAMBIA1391 vectors, each having a GUS reporter gene. GUS staining was performed in GUS staining buffer (50 mM phosphate buffer (pH 7.2), 0.1% (v/v) Triton™ X-100, 0.5 mM K_4_Fe(CN)_6_H_2_O, 0.5 mM K_3_Fe(CN)_6_, and 0.5 mM X-Gluc) at 37 °C in the dark. Then, 100% ethanol was applied to decolorize the tissues. The images were captured by utilizing a ZEISS SV11 stereoscope.

### Dual-luciferase reporter assays

Transient expression assays were carried out using tobacco (*N. benthamiana*) leaves. The promoter fragments of *LcACO2/3* and *LcACS1/4* were subcloned into pGreenII 0800-LUC to generate reporter constructs (Fig. S2). The effectors (35 S:LcBZR1/2) were generated by recombining the *LcBZR1*/*2* genes into the empty pGreenII 62-SK vector. The effector and reporter constructs were coinjected into tobacco leaves with or without ET (50 µL L^–1^) for 48 h. The activities of LUC and REN luciferases were detected by a Luminoskan Ascent Microplate Luminometer (Thermo Fisher Scientific, Waltham, MA, USA) and the Dual-Luciferase^®^ Reporter Assay System (YEASEN, Shanghai), and the values of LUC/REN were calculated. At least six independent biological replicates were examined.

### Electrophoretic mobility shift assay

The fusion proteins LcBZR1–GST and LcBZR2–GST were generated through prokaryotic expression in vitro. The coding sequences of *LcBZR1* and *LcBZR2* were each fused into a pGEX-4T-1 vector encoding a GST tag to generate the recombined vectors. Then, the recombinant vectors were expressed in *Escherichia coli* BL21 (DE3). Next, 1 mM isopropyl β-d-1-thiogalactopyranoside was used to induce protein production. GSTSep Glutathione Agarose Resin (YEASEN, Shanghai) was used to purify the fusion proteins. Electrophoretic mobility shift assays (EMSAs) were carried out by utilizing a LightShift Chemiluminescent EMSA Kit (Thermo Fisher Scientific). Briefly, the GST protein alone was used as the negative control. DNA fragments with biotin labels were prepared and used as probes, with unlabeled DNA with the same sequences being used as competitors.

### Data analysis

All experimental data are presented as the averages of three or six independent biological replicates. Statistical significance between samples was investigated by Student’s *t* test or Duncan’s test.

### Primers

All primers listed in Table S1 were produced by using Primer 3 (http://bioinfo.ut.ee/primer3/).

## Supplementary information

Figure S1

Figure S2

Table S1
