# Supplementary figures and images for: Brassinosteroids suppress ethylene-induced fruitlet abscission through LcBZR1/2-mediated transcriptional repression of LcACS1/4 and LcACO2/3 in litchi
Source: Hortic Res. 2021 May 1;8:105. doi: 10.1038/s41438-021-00540-z (PMC8087802; doi:10.1038/s41438-021-00540-z)

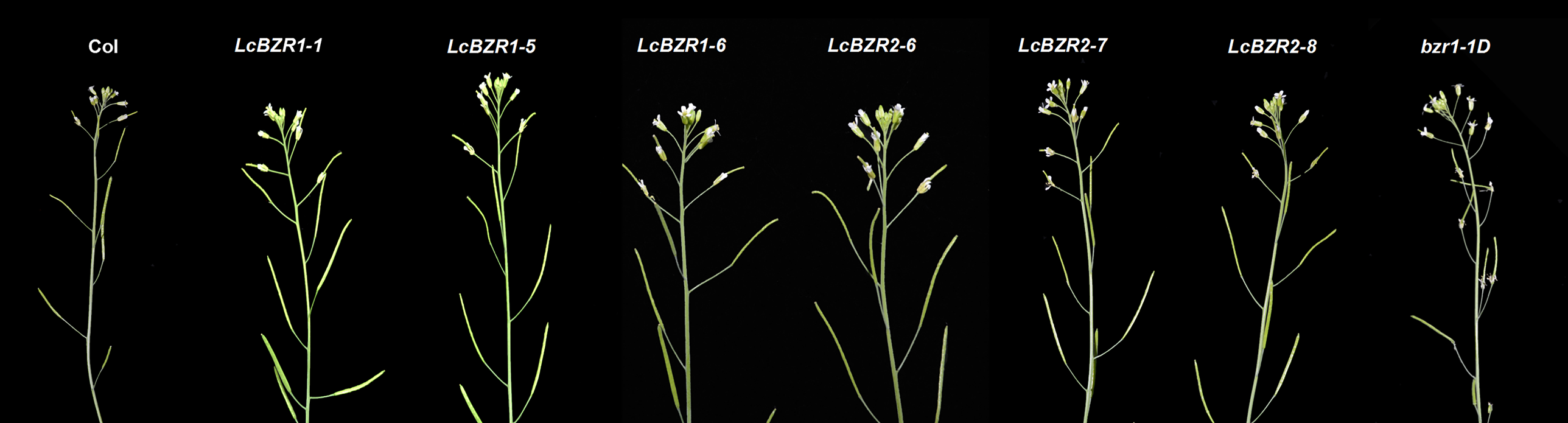

Supplement: Supplementary file 1 — Figure S1 [file 41438_2021_540_MOESM1_ESM.jpg]

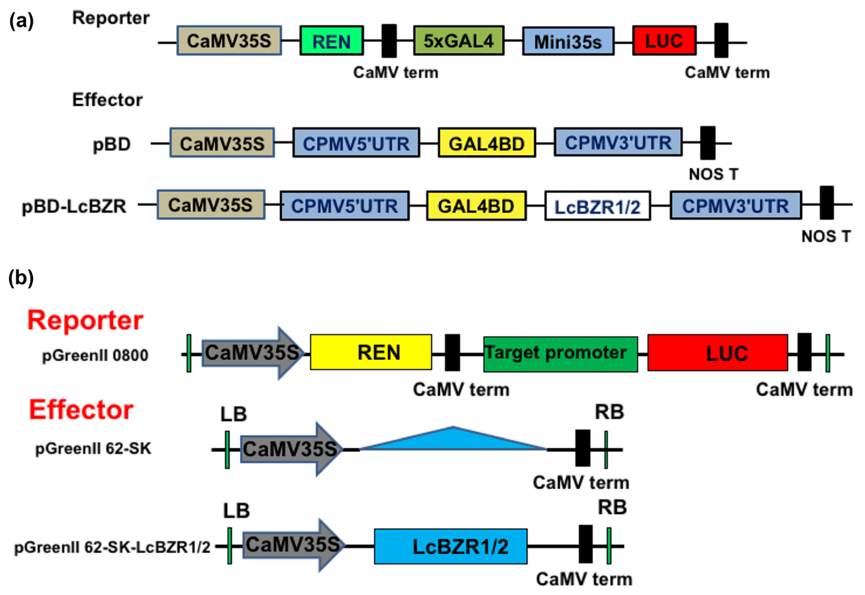

Supplement: Supplementary file 2 — Figure S2 [file 41438_2021_540_MOESM2_ESM.png]
