# Supplementary material for: Brassinosteroids suppress ethylene-induced fruitlet abscission through LcBZR1/2-mediated transcriptional repression of LcACS1/4 and LcACO2/3 in litchi
Source: Hortic Res. 2021 May 1;8:105. doi: 10.1038/s41438-021-00540-z (PMC8087802; doi:10.1038/s41438-021-00540-z)
Supplement: Supplementary file 3 — Table S1 [file 41438_2021_540_MOESM3_ESM.pdf]

Table S1 Summary of primers used in this study

| Assay | Primer sequence (5’-3’) | Restriction Site |
|---|---|---|
| Subcellular localization | *LcBZR1-GFP-F* : caaattcgcgaccggtATGACATCAGGAGGATCAGGGAGGTTAC | *Age* I |
| | *LcBZR1-GFP-R* : tgctagtcataccggtTGCGCGCGTCTTGGTAGTGCCAAGTGT | |
| | *LcBZR2-GFP-F* : caaattcgcgaccggtATGACGTCAGATGGGGCGACCTCGACGT | |
| | *LcBZR2-GFP-R* : tgctagtcataccggtAACCTGAGTCTTCCCGGTTCCAAGTGTG | |
| qRT-PCR | *LcBZR1-qF*: CCCCTCCTTCGCCTACCTTTA | |
| | *LcBZR1-qR*: CTCACCTTCCCACGGTTTCAC | |
| | *LcBZR2-qF*: CAACTTCGCCAACCTTTAATCT | |
| | *LcBZR2-qR*: CATTCCTTCCATTCCTACCTCA | |
| | *LcACO2-qF* : AAGTCAGTAACTACCCTCCATGCCCTAA | |
| | *LcACO2-qR* : GCCATCTGGTTGAGCAATCACACGG | |
| | *LcACO3-qF* : TGTCCTCATCCCGACCTTGTAAACG | |
| | *LcACO3-qR* : GGTAGCATTAACACGGTGCCAGAC | |
| | *LcACS1-qF* : CTGACCGCATTGTTATGAGTGGTGGAG | |
| | *LcACS1-qR* : GAGCCTTTTCATATGCAGCTTCCAA | |
| | *LcACS4-qF* : ACATATGGAGCCGCATTCACGTTG | |
| | *LcACS4-qR* : AGCACCTGATTCCTGCCGATTT | |
| | *AtACO4-qF* : GCGGCATCATCCTCCTCTT | |
| | *AtACO4-qR* : GTACTTCCCATTGGTTATCACCTC | |
| | *AtACS1-qF* : ATGTCTCAGGGTGCATGTGA | |
| | *AtACS1-qR* : CCGCCGTACAAATAGATGCC | |
| | *LcEF-1α-qF* : CGTGGATTCGTCGCATCTAAC | |
| | *LcEF-1α-qR* : CCGCCTGTCAATCTTGGTCAT | |
| | *AtUBQ-qF* : GATCTTTGCCGGAAAACAATTGGAGGATGGT | |
| | *AtUBQ-qR* : CGACTTGTCATTAGAAAGAAAGAGATAACAGG | |
| Transgenic assay | *LcBZR1-35SF* : ggactcttgaccatggtaATGACATCAGGAGGATCAGGGAGGTTAC | *Nco* I |
| | *LcBZR1-35SR* : gtcagatctaccatggtTGCGCGCGTCTTGGTAGTGCCAAGTGT | |
| | *LcBZR2-35SF* : ggactcttgaccatggtaATGACGTCAGATGGGGCGACCTCGACGT | |
| | *LcBZR2-35SR* : gtcagatctaccatggtAACCTGAGTCTTCCCGGTTCCAAGTGTG | |
| GUS assay | *Pro*$_{LcBZR1}$*::GUS-F:* tcgacggatccccgggaattcATTTTCTCAACCCGCCCAAGTCATG | *EcoR* I |
| | *Pro*$_{LcBZR1}$*::GUS-R*: gtggactcctcttagaattcCCTCCAACACAAAACCCTTCTCTCT | |
| | *Pro*$_{LcBZR2}$*::GUS-F*: tcgacggatccccgggaattcTTGAGACAAAGTAAACAACAGAGCG | |
| | *Pro*$_{LcBZR2}$*::GUS-R*: gtggactcctcttagaattcCCGACACCTGAAACTCAATCAATCT | |
| EMSA assay | *pGEX-4T-1-LcBZR1-F* : gaattcccgggtcgacATGACATCAGGAGGATCAGGGAGGTTAC | *Sal* I |
| | *pGEX-4T-1-LcBZR1-R* : ggccgctcgagtcgacTGCGCGCGTCTTGGTAGTGCCAAGTGT | |
| | *pGEX-4T-1-LcBZR2-F* : gaattcccgggtcgacATGACGTCAGATGGGGCGACCTCGACGT | |
| | *pGEX-4T-1-LcBZR2-R* : ggccgctcgagtcgacAACCTGAGTCTTCCCGGTTCCAAGTGTG | |
| | *LcACO2-probe-F* : TAAAGAGAGATAAACTCAATAGATcacacgCGGGCTTAAACCACCAGACAA | |
| | *LcACO2-probe-R* : TTGTCTGGTGGTTTAAGCCCGcgtgtgATCTATTGAGTTTATCTCTCTTTA | |
| | *LcACO2-mprobe-F* : TAAAGAGAGATAAACTCAATAGATaaaaaaCGGGCTTAAACCACCAGACAA | |
| | *LcACO2-mprobe-R* : TTGTCTGGTGGTTTAAGCCCGttttttATCTATTGAGTTTATCTCTCTTTA | |
| | *LcACO3-probe-F* : ACTTTTTGATCACGAAAATTGGcaaatgTTGCCAAGTAC | |
| | *LcACO3-probe-R* : GTACTTGGCAAcatttgCCAATTTTCGTGATCAAAAAGT | |
| | *LcACO3-mprobe-F* : ACTTTTTGATCACGAAAATTGGggggggTTGCCAAGTAC | |

| | | |
|---|---|---|
| | *LcACO3-mprobe-R* : GTACTTGGCAAccccccCCAATTTTCGTGATCAAAAAGT | |
| | *LcACS1-probe-F* : TATTTACATTTTTAACATTTTCcacacgTCCACCAAGACCCACTAGTT | |
| | *LcACS1-probe-R* : AACTAGTGGGTCTTGGTGGAcgtgtgGAAAATGTTAAAAATGTAAATA | |
| | *LcACS1-mprobe-F* : TATTTACATTTTTAACATTTTCaaaaaaTCCACCAAGACCCACTAGTT | |
| | *LcACS1-mprobe-R* : AACTAGTGGGTCTTGGTGGAttttttGAAAATGTTAAAAATGTAAATA | |
| | *LcACS4-probe-F* : CAAGTACCCCTCTCTTCTTCAAGGTATcgtgcgTGTATGGACAATTTTGTT | |
| | *LcACS4-probe-R* : AACAAAATTGTCCATACAcgcacgATACCTTGAAGAAGAGAGGGGTACTTG | |
| | *LcACS4-mprobe-F* : CAAGTACCCCTCTCTTCTTCAAGGTATaaaaaaTGTATGGACAATTTTGTT | |
| | *LcACS4-mprobe-R* : AACAAAATTGTCCATACAttttttATACCTTGAAGAAGAGAGGGGTACTTG | |
| Dual-luciferase transient expression assay | *LcBZR1-pBD-F* : tcgccgaccggtaggcctATGACATCAGGAGGATCAGGGAGGTTAC | *Stu* I |
| | *LcBZR1-pBD-R* : aaccagagttaaaggcctTGCGCGCGTCTTGGTAGTGCCAAGTGT | |
| | *LcBZR2-pBD-F:* tcgccgaccggtaggcctATGACGTCAGATGGGGCGACCTCGACGT | |
| | *LcBZR2-pBD-R:* aaccagagttaaaggcctAACCTGAGTCTTCCCGGTTCCAAGTGTG | |
| | *LcBZR1-pGreenII 62-SK-F* : tagaactagtggatccATGACATCAGGAGGATCAGGGAGGTTAC | *Bam*HI *Hind*III |
| | *LcBZR1-pGreenII 62-SK-R* : cggtatcgataagcttTGCGCGCGTCTTGGTAGTGCCAAGTGT | |
| | *LcBZR2-pGreenII 62-SK-F* : tagaactagtggatccATGACGTCAGATGGGGCGACCTCGACGT | |
| | *LcBZR2-pGreenII 62-SK-R* : cggtatcgataagcttAACCTGAGTCTTCCCGGTTCCAAGTGTG | |
| | *Pro* $_{LcACO2}$ *-LUC-F* : tatagggcgaattggAAGGAAGATTTGTTTCATAATAAAGTTAGAATTATTATTA | *Kpn I* *Nco I* |
| | *Pro* $_{LcACO2}$ *-LUC-R* : ttggcgtcttccatggTGTTGTTGCTGTTGTTGTATTAGTTTTCTTG | |
| | *Pro* $_{LcACO3}$ *-LUC-F* : tatagggcgaattggTCTAGCATGTTAAGAGCATTGCCAAATA | |
| | *Pro* $_{LcACO3}$ *-LUC-R* : ttggcgtcttccatggCTTTCTCTTGTTTTCTTTCTATAATATTGTAAAGGT | |
| | *Pro* $_{LcACS1}$ *-LUC-F* : tatagggcgaattggGTCAATCCAGGCTGGACTGGTTGA | |
| | *Pro* $_{LcACS1}$ *-LUC-R* : ttggcgtcttccatggCTTTGTGCTTCTAAAAGGTCTTGAATGTAT | |
| | *Pro* $_{LcACS4}$ *-LUC-F* : tatagggcgaattggATACAATTGAAGTTATCACTAGATATAAGTCATTG | |
| | *Pro* $_{LcACS4}$ *-LUC-R* : ttggcgtcttccatggTTTGACACCCACCAAGCTATCTAGC | |
